# Process Simulation of 1,4-Cyclohexanedimethanol Production from Waste PETs

**DOI:** 10.1021/acsomega.6c01699

**Published:** 2026-05-11

**Authors:** Berk Esin, Serhat Sezer, Gülhayat Nasun-Saygılı

**Affiliations:** Chemical Engineering Department, 52971 Istanbul Technical University, 34469 İstanbul, Turkey

## Abstract

The increasing accumulation of plastic waste, particularly poly­(ethylene terephthalate) (PET), necessitates sustainable recycling strategies. This study presents a process design for producing 1,4-cyclohexanedimethanol (CHDM) via chemical recycling of post-consumer PET using bis­(2-hydroxyethyl) terephthalate (BHET) as an intermediate. The proposed process includes PET glycolysis followed by BHET hydrogenation and was modeled and simulated using Aspen Plus and Aspen EDR. A production capacity of 7920 tons of PET annually was selected, resulting in 5000 tons/year of CHDM with 97.4% purity. Thermodynamic models (POLYUF, POLYSL, and NRTL-HOC) were applied across various process stages. Kinetic parameters were derived from the literature and simulation databases. Equipment sizing, energy integration, and economic analysis were performed, and the total production cost was estimated at 46.7 million USD. A profitability analysis via CAPCOST revealed a net present value of 4.39 million USD, a discounted cash flow rate of return of 16.14%, and a discounted payback period (DPBP) of 3.8 years. These results indicate that CHDM production from PET waste is both technically and economically viable, providing an environmentally friendly valorization pathway for plastic waste.

## Graphical abstract

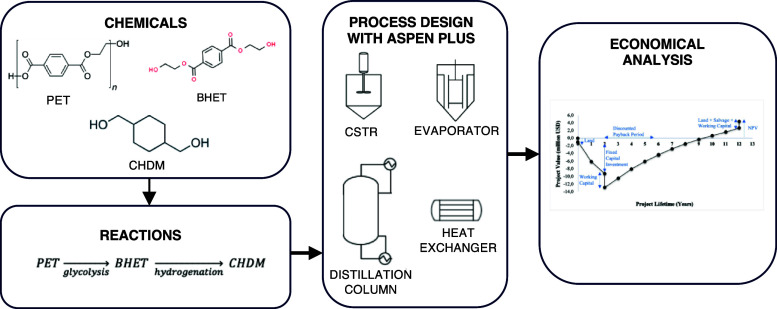

## 1 Introduction

Since the development of synthetic polymers derived from petrochemical sources, the plastics industry has experienced tremendous growth due to the desirable properties of plastics such as durability, lightweight, and cost efficiency.^1^ These properties have enabled plastics to become indispensable across various sectors, including packaging, construction, automotive, and electronics. As a result, global plastic production reached 413.8 million tons in 2023, with Europe alone contributing 54 million tons.^2^

Among the plastic types, polyethylene terephthalate (PET) plays a significant role in industrial applications. PET is a thermoplastic polymer belonging to the polyester family^3^ and is widely used in products such as beverage bottles, electronic components, automotive parts, sports equipment, and packaging films. Early applications of PET included fibers and films, with the latter being used in photography, X-ray imaging, and food packaging. PET also functions as an effective electrical insulator and can be produced with different mechanical and thermal properties based on its target application area.^4^

Global plastic production increased substantially between 1950 and 2015, reaching 407 million tons in 2015. Of this total, 33 million tons consisted of PET. This sharp rise in plastic production was accompanied by a corresponding increase in plastic waste generation, which amounted to 302 million tons in 2015 including 32 million tons of PET waste.^5^

The cumulative plastic waste generation and disposal trends between 1950 and 2050 highlight the growing environmental burden of plastic consumption.^5^ The total primary plastic waste generated is projected to reach approximately 25,000 million metric tons by 2050, indicating an exponential increase over the century. Despite ongoing efforts, the majority of this waste is still either discarded or incinerated, while only a relatively small fraction is recycled. Notably, the gap between generated and recycled waste continues to widen, emphasizing the insufficiency of the current recycling infrastructure. This imbalance underscores the necessity of advancing chemical recycling technologies, such as PET glycolysis and 1,4-cyclohexanedimethanol (CHDM) production, which offer higher efficiency and better material recovery compared with conventional mechanical recycling. Adopting such methods is vital to mitigate the long-term accumulation of plastic waste and reduce greenhouse gas emissions associated with plastic disposal.

Despite the scale of plastic production and consumption, only 18% of global plastic waste was recycled, while 58% was landfilled.^6^ Coastal countries contribute an estimated 4.8–12.7 million tons of plastic waste to the oceans annually, posing serious threats to marine ecosystems. Furthermore, approximately 95% of plastic packaging is used only once, leading to massive material and economic losses.^7^ The incineration of plastic waste, which accounts for around 12% of municipal solid waste, releases toxic pollutants including dioxins, mercury, and furans, contributing significantly to air pollution and public health risks.^8^

In addition to environmental concerns, plastic production significantly contributes to greenhouse gas emissions. Plastics manufacturing was responsible for 1.78 gigatons of CO_2_ emissions, a number projected to rise to 6.5 gigatons by 2050 if current production trends continue.^6^ In light of these environmental and climatic issues, the European Commission’s Circular Economy Action Plan has proposed recycling 55% of plastic packaging waste by 2030. Furthermore, the European Green Deal’s “Zero Pollution” target and the Chemicals Strategy for Sustainability promote advanced solutions such as chemical recycling and smart waste management.^9,10^

Among the different recycling strategies, chemical recycling is particularly valuable for PET waste. Unlike mechanical methods, it enables depolymerization back to monomers, thus allowing for the production of high-purity raw materials suitable for use in virgin-quality plastics or other valuable chemicals. Several chemical recycling techniques for PET have been studied including hydrolysis, methanolysis, aminolysis, and glycolysis. The selection of the appropriate method depends on the desired monomer product and reaction conditions.^4^

One of the most prominent intermediates in PET chemical recycling is bis­(2-hydroxyethyl) terephthalate (BHET), which can be produced via the glycolysis of PET. BHET is a commercially available monomer with a structure similar to PET itself and is extensively used in research and industrial applications related to polyester synthesis.^11^ It is commonly used as a raw material in PET manufacturing plants that utilize either terephthalic acid (TPA) or dimethyl terephthalate (DMT).^12^ BHET also finds applications in the production of textile softeners, polyurethane foams, and unsaturated polyester resins, especially in polyester–polyol copolymers. BHET can be synthesized by the transesterification of DMT with ethylene glycol (EG), direct esterification of TPA with EG, or the glycolysis of PET waste, as illustrated in Figure 1.^13^

**Figure 1:**
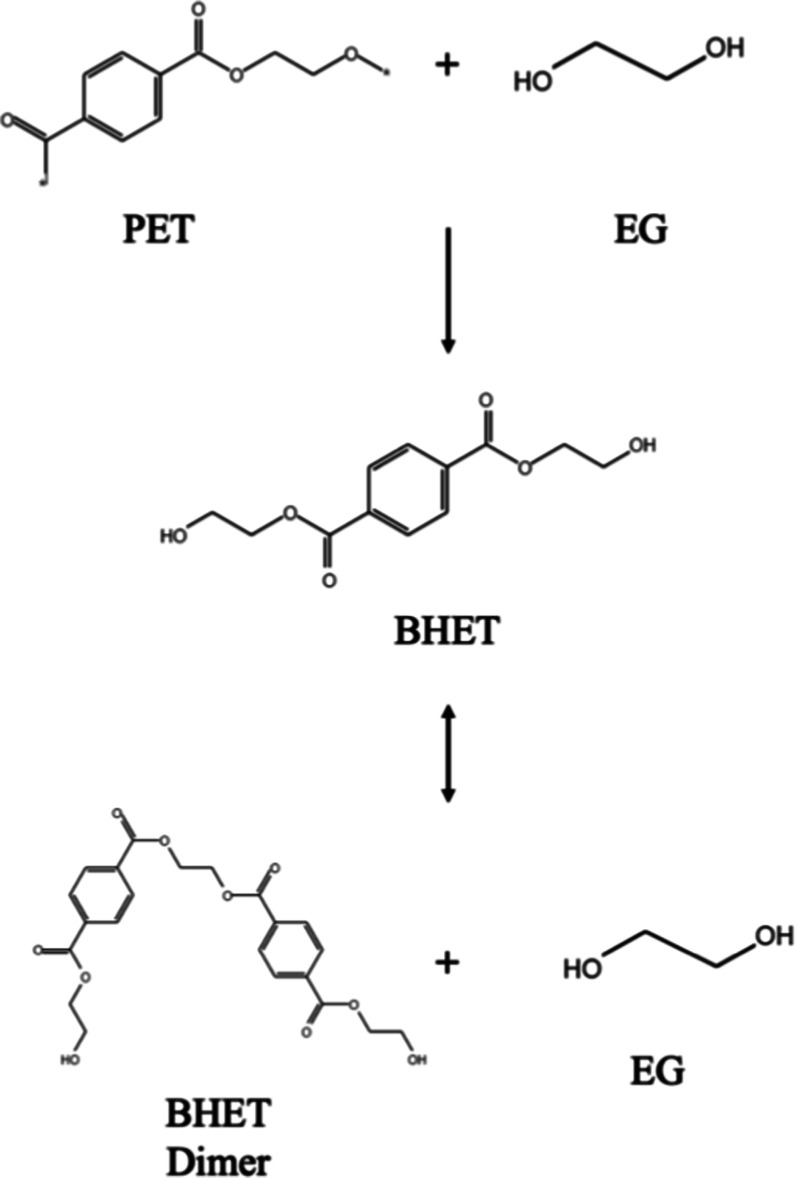
Reaction scheme of BHET and byproduct formation via PET glycolysis.

An important end-product of PET chemical recycling is cyclohexanedimethanol (CHDM), a key monomer used in the production of specialty polyesters with improved chemical resistance, transparency, and thermal stability.^14^ CHDM-containing copolyesters such as PCT, PCTG, PETG, and PCTA exhibit enhanced properties compared to conventional PET, including higher melting points and superior mechanical performance.^15^ These copolyesters are widely used in applications requiring high clarity and durability, such as medical packaging and optical films.

Traditionally, CHDM is synthesized via a two-stage hydrogenation of DMT, involving palladium and copper-chromite catalysts under high temperature and pressure.^16^ However, an alternative and potentially more sustainable pathway involves a single-step hydrogenation of BHET using a trimetallic RuPtSn/Al_2_O_3_ catalyst. As demonstrated in a study, under optimized conditions of 2 h at 170 °C followed by 6 h at 260 °C under 5 MPa hydrogen pressure, BHET was converted with 100% efficiency and yielded 87.1% CHDM.^17^

Recent literature has further underscored the potential of this pathway. For instance, Mo et al.^18^ reported a tandem reaction strategy for upcycling PET wastes into high-value-added CHDM, while Huang et al.^19^ investigated the direct valorization of waste polyester for CHDM production. Furthermore, the economic and environmental implications of circularizing CHDM-containing materials (PET-G) have recently been evaluated via life cycle assessment and techno-economic analysis.^20^

Although the depolymerization of PET to BHET has been the subject of various simulation studies,^21–23^ these works generally conclude at the monomer recovery stage or primarily compare purification methods such as crystallization versus evaporation. There is a noticeable gap in the literature regarding the simulation of an integrated, continuous process that extends beyond BHET recovery to the production of high-value chemicals, such as 1,4-cyclohexanedimethanol (CHDM), from waste sources. Furthermore, detailed strategies for handling byproducts, such as the separation and recycling of BHET dimers within a continuous loop, are often overlooked. This study aims to fill this gap by presenting a complete “waste to product” process design converting post-consumer PET directly into high-purity CHDM and evaluating its technical and economic feasibility under optimized industrial conditions.

In this study, the production of CHDM from PET-derived BHET was simulated by using Aspen Plus process modeling software. The simulation results were analyzed to assess the feasibility of implementing this process on an industrial scale, focusing on both technical performance and environmental sustainability.

## 2 Simulation

Process Options and Selection: CHDM can be synthesized via two main methods: (1) through hydrogenation of DMT and (2) through hydrogenation of bis­(2-hydroxyethyl) terephthalate (BHET). In this study, the BHET route was selected due to its environmentally friendly nature and economic feasibility. Specifically, CHDM production from BHET derived via glycolysis of post-consumer PET waste was preferred over the DMT route, as the BHET method requires lower capital investment, consumes less energy, and operates under milder reaction conditions.^24,25^ Additionally, in the process of BHET production from PET, two different methods—evaporation and crystallization—are available for EG separation.^26–28^ Although BHET recovery is slightly higher via evaporation, crystallization generates considerable wastewater, complicating treatment.^29^ Therefore, the evaporation method was chosen for the glycol separation in this process. A simplified block flow diagram validating the selected process route is illustrated in Figure 2.

**Figure 2:**
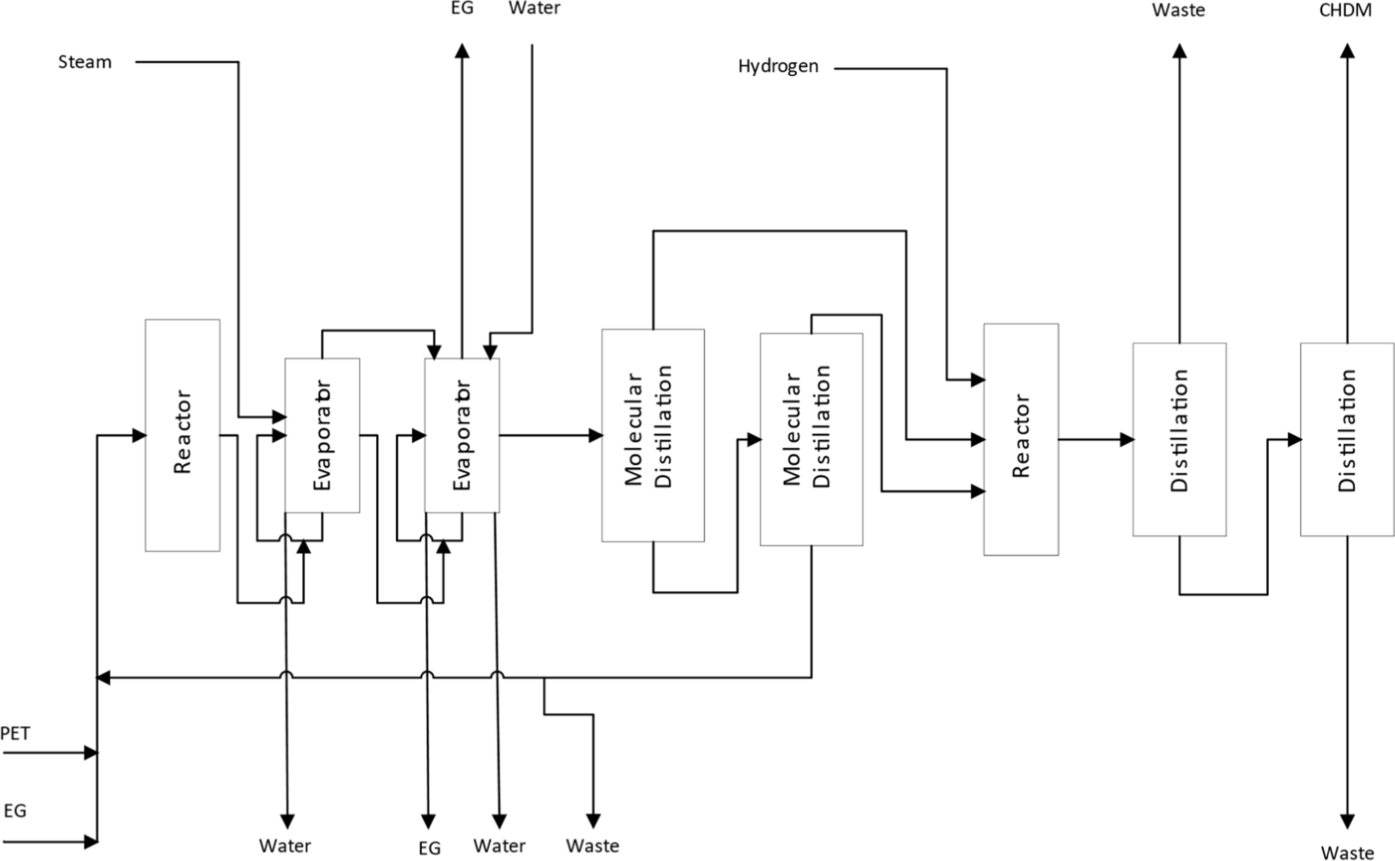
Simplified block flow diagram of the CHDM production process.

Capacity Selection: According to studies using data from PAGEV and TÜİK, PET recycling in Turkey shows an increasing trend—from 0.155 million tons in 2022 to a projected 0.169 million tons by 2030. In comparison, landfilled PET was reported as 0.344 million tons in 2022 and is projected to increase to 0.373 million tons by 2030. Furthermore, PET recycling is considered a superior alternative to incineration for energy recovery.^30^ Based on this information, the capacity for this process was determined as 7920 tons of PET recycling annually.

Due to the projected increase in PET bottle consumption in Turkey, it is anticipated that the volume of PET being landfilled will also rise. To make efficient use of this increasing PET waste stream, we selected a continuous process configuration. In this process, the main raw materials are post-consumer PET particles and EG, while hydrogen gas is utilized for the hydrogenation of BHET, which is obtained through glycolysis. For thermal energy needs, steam at both low pressure (5 barg) and high pressure (41 barg) is used, whereas cooling is carried out using water at 28 °C. The plant location was chosen to be in Çorlu, Tekirdağ, Türkiye due to its proximity to a major urban area where PET consumption is high and to facilitate import and export operations logistically. The glycolysis reactions occurring in the first reactor are represented in Reactions R1 and R2, where Reaction R1 is chemical recycling of PET into the BHET monomer, and Reaction R2 is dimerization of BHET into the BHET dimer.

R1 $$PET+C_{2}H_{6}O_{2}\overset{k_{1}}{\to }C_{12}H_{14}O_{6}$$

R2 $$2C_{12}H_{14}O_{6}\overset{k_{2}/k_{-2}}{↔}C_{22}H_{22}O_{10}+C_{2}H_{6}O_{2}$$

The kinetic parameters of the reactions are listed in Table 1.

**Table 1:** Kinetic Parameters

reaction	*E*_*i*_ (kcal/mol)	*A*_*i*_ (m^3^/kmol·s)	*K*
R1	11.04 ^31^	1163.05 ^31^	-
R2	18.5 ^32^	11,333.3 ^32^	1.13 ^33^

The reaction kinetic parameters provided in Table 1 are defined by the rate expressions given in eqs 1–4:

1 $${-r}_{1}=A_{1}\;\exp\left(-\frac{E_{1}}{RT}\right)C_{PET}C_{EG}$$

2 $${-r}_{2}=A_{2}\;\exp\left(-\frac{E_{2}}{RT}\right)C_{BHET}^{2}$$

3 $$r_{-2}=A_{-2}\;\exp\left(-\frac{E_{-2}}{RT}\right)C_{BHET\;dimer}C_{EG}$$

4 K=k2k−2=A2exp(−E2RT)A−2exp(−E−2RT)

The *E*_2_ value was determined using data from the Aspen Plus software database and the Joback method.^34^ After obtaining the *E*_2_, the corresponding *A*_2_ value was calculated using eq 4. Additionally, during PET glycolysis in the first reactor, EG, similar to BHET, tends to undergo dimerization. However, since this occurs in negligible amounts, the dimerization of EG was not taken into consideration.^23^ The BHET hydrogenation reactions that occur in the second reactor are presented in Reactions [Disp-formula fdR3]–[Disp-formula fdR10]:

R3 $$C_{12}H_{14}O_{6}+{3H}_{2}\to C_{12}H_{20}O_{6}$$

R4 $$C_{12}H_{20}O_{6}+{4H}_{2}\to C_{8}H_{16}O_{2}+2C_{2}H_{6}O_{2}$$

R5 $$C_{12}H_{20}O_{6}+{6H}_{2}\to C_{8}H_{16}O_{2}+2H_{2}O+2C_{2}H_{6}O$$

R6 $$C_{12}H_{14}O_{6}+{4H}_{2}\to C_{10}H_{12}O_{2}+C_{2}H_{6}O_{2}+2H_{2}O$$

R7 $$C_{10}H_{12}O_{2}+{2H}_{2}\to C_{8}H_{10}+C_{2}H_{6}O_{2}$$

R8 $$C_{8}H_{16}O_{2}+H_{2}\to C_{8}H_{16}O+H_{2}O$$

R9 $$C_{8}H_{16}O+H_{2}\to C_{8}H_{16}+H_{2}O$$

R10 $$C_{22}H_{22}O_{10}+{6H}_{2}\to C_{22}H_{34}O_{10}$$

The second reactor was simulated in Aspen Plus based on the experimental conversion rates provided in Table 2.^17^

**Table 2:** Conversion Rates

reaction	conversion rate	reference chemical
R3	0.954	BHET
R4	0.9	BHCD
R5	1	BHCD
R6	1	BHET
R7	0.8	ethyl *p*-toluate
R8	0.083	CHDM
R9	0.8	*trans*-1,4-dimethylcyclohexane
R10	1^a^	C_22_H_34_O_10_

a It has been assumed that, in the BHET dimer, only the benzene rings are hydrogenated into cyclohexane rings.

In this study, three different thermodynamic models were employed to accurately represent the phase behavior throughout the process. Due to the lack of experimental data for the glycolysis stage, the POLYUF (Polymer UNIFAC) model was used to estimate phase equilibria.^35^ In light of the high-pressure operation and the presence of supercritical fluids in the hydrogenation reactor, the POLYSL (Polymer Sanchez–Lacombe) model was employed for this unit, as it has been demonstrated to provide accurate and reliable predictions under similar conditions.^36^ Following the hydrogenation stage, two distillation columns are employed. Since these columns operate at relatively low pressures but at temperatures exceeding 200 °C under which EG dimerization may occur,^37^ the NRTL-HOC model was selected to accurately represent the non-ideal behavior of the mixture.^35^ Regarding the simulation and process structure, in Aspen Plus, the PET polymer, BHET, and BHET dimer were defined as oligomers. To enable this representation, T-EG, B-EG, and B-TPA were introduced as segment units, where “B” indicates a repeating segment, and “T” represents a terminal segment. BHET was described in the system as consisting of 2 T-EG and 1 B-TPA segments, while the BHET dimer was defined as comprising 2 T-EG, 1 B-EG, and 2 B-TPA segments. The PET polymer was defined with segmental mole fractions of 0.5 B-EG and 0.5 B-TPA, and its number-average molecular weight was taken as 20,000 g/mol.^38^ All other chemicals were defined as conventional components within Aspen Plus. Furthermore, the BHET dimer, BHCD, and C_22_H_34_O_10_ were not available in the Aspen Plus database and were therefore manually added into the system, and their properties were estimated by Aspen Plus and the Joback method. While general equipment sizing was performed within Aspen Plus, the detailed design of heat exchangers was carried out using Aspen Exchanger Design & Rating (EDR). Specifically, the allocation of fluids to the shell or tube side was determined by Aspen EDR software, which optimized the configuration based on rigorous thermal and hydraulic analysis to ensure convergence and operational feasibility.

## 3 Results and Discussion

The process flow diagram for CHDM production is presented in Figure 3. The CHDM production process was designed based on the annual recycling of 7920 tons of PET. To achieve high PET conversion during glycolysis, excess EG is fed into the system. The PET-to-EG mass ratio was set at 1:5, and the catalyst (Zn^2+^)/PET ratio was set at 0.01. This mixture, at 25 °C and 1.013 bar with a total flow rate of 6000 kg/h, is fed into the glycolysis reactor (R-101) along with stream 23 (145.23 kg/h, containing 42.5 wt % BHET and 57.5 wt % BHET dimer). The glycolysis reactor (R-101) operates isothermally at 190 °C and 1.013 bar, achieving nearly 100% PET conversion. Unreacted PET (0.74 kg/h) is removed from the system via a filter (F-101).

**Figure 3:**
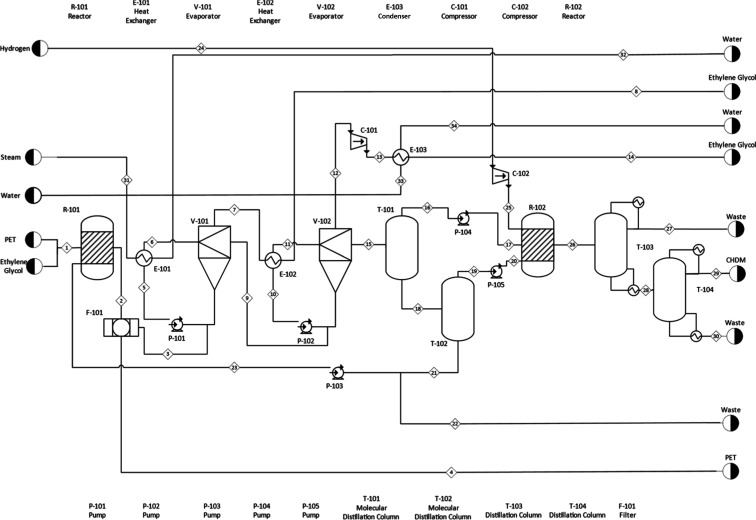
Process flow diagram.

Following the glycolysis step, in order to remove the excess EG introduced into the reactor, the stream is directed to a double-effect evaporator system. Stream 3 from the filter unit (F-101) enters the suction line of the pump (P-101) associated with the first evaporator (V-101), and the resulting liquid phase is subsequently fed into the suction line of the pump (P-102) connected to the second evaporator (V-102). The first evaporator (V-101) operates at 0.01 bar and 90.1 °C, while the second (V-102) operates at 0.00031 bar and 74.2 °C. For the circulation in V-101, a heat exchanger (E-101) increases the temperature by 3 °C using 5 barg of steam at 307 kg/h. In V-102, the circulation is heated using a heat exchanger (E-102) by reusing 2273.02 kg/h stream 7, which contains vaporized EG from V-101. The EG separated from the second evaporator is compressed in a compressor (C-101) to 0.01 bar and then sent to a condenser (E-103), where it is condensed using 71,019 kg/h of cooling water at 28 °C. In total, 4631.14 kg/h of EG is removed from the system via this double-effect evaporator setup.

Following this step, to separate the BHET dimer formed as a byproduct in the glycolysis reactor (R-101) and recycle it, two molecular distillation columns were used. The first column (T-101) operates at 0.00023 bar and 205 °C, and the second (T-102) operates at 0.00015 bar and 208 °C. Of the 149.63 kg/h of BHET dimer fed into T-101, 56.4% is separated and recycled back to the glycolysis reactor via T-102. A 1.47 kg/h stream (stream 22) is purged to prevent accumulation. The distillate streams from the two molecular distillation columns (streams 16 and 19) are sent to the hydrogenation reactor (R-102) via pumps P-104 and P-105. Additionally, 73.59 kg/h of hydrogen is compressed in a compressor (C-102) to 50 bar and fed into the reactor.

The hydrogenation reactor (R-102) is operated under isothermal conditions at 50 bar and 260 °C, using a trimetallic catalyst characterized by a Ru/Sn molar ratio of 1.5. In this reactor, both BHET and the BHET dimer undergo complete conversion. To purify CHDM, the main product, from byproducts, two distillation columns are used. The first column (T-103) contains 8 trays, with a condenser pressure of 0.902 bar and a pressure drop of 0.0101 bar per tray. The bottom product from T-103 is fed into the second column (T-104), which has 10 trays, a condenser pressure of 0.405 bar, and the same per-tray pressure drop. The distillate from T-104 yields 631.25 kg/h of CHDM with 97.4 wt % purity. Streams 22, 27, and 30 are purged from the system as waste. A more detailed representation of the streamflow, including all connections and compositions, is provided in Table S1.

## 4 Economic Analysis

The economic evaluation of the process, including both production costs and profitability analysis, was conducted using the CAPCOST program.^39^ All associated costs involved in the production of CHDM were considered to estimate the total production cost (COM), which incorporates elements such as fixed capital investment (FCI), operational labor cost (COL), utility cost (CUT), raw material cost (CRM), and land cost. Since the cleansing and shredding of waste PET are not modeled in Aspen Plus, the price of clean PET flakes was used as the basis for the calculation. The revenue is generated from both CHDM production and the recovery of EG. Following the calculation of the production cost, a profitability analysis was performed. The depreciation-free cost of manufacture (COMd) was calculated using CAPCOST software, and the parameters used in this computation are provided in Table 3. As a result, the total production cost was estimated to be 46,730,272 USD. Detailed cost estimation tables taken by CAPCOST software are provided in the Supporting Information (Tables S5–S8).

**Table 3:** Production Cost Parameters and Total Manufacturing Cost

FCI	9,380,000 USD
*C*_RM_	34,543,600 USD
*C*_UT_	148,936 USD
*C*_OL_	280,800 USD
*C*_WT_	1,296,720 USD
COM_d_	46,730,272 USD

The profitability analysis was conducted using both discounted and non-discounted evaluation methods via CAPCOST software. The non-discounted methods do not take into account the time value of the money and are not recommended for evaluating new, large projects. Therefore, we will evaluate our process using the discounted method.

As illustrated in Table 4, the discounted net present value (NPV) was calculated as 4.39 million USD, with a discounted cash flow rate of return (DCFROR) of 16.14% and a discounted payback period (DPBP) of 3.8 years. In contrast, the non-discounted method yielded a capital cost (CCP) of 20.72 million USD, a return on investment (ROROI) of 22.09%, and a simple payback period (PBP) of 2.8 years.

**Table 4:** Comparison of Profitability Criteria

discounted		non-discounted	
NPV (million)	4.39	CCP (million)	20.72
DCFROR (%)	16.14	ROROI (%)	22.09
DPBP (year)	3.8	PBP (year)	2.8

The payback period and discounted payback period are key criteria for assessing the profitability of a project. Since the discounted profitability indicators are considered in this study, the discounted payback period was determined to be 3.8 years. The NPV was calculated as 4.39 million USD, and the DCFROR was found to be 16.14%. These values clearly indicate that the project is economically viable. Cash Flow Analysis: A cash flow diagram represents the cash flow over a time scale in a graphical format. The cash flow diagram for CHDM production, generated using CAPCOST software, is shown in Figure 4. The tax rate and annual interest rate of 30% and 10%, respectively, were applied. The construction period was assumed to be 2 years, and the project lifetime after startup was taken as 10 years, resulting in a total project lifespan of 12 years. The MACRS depreciation method was chosen and applied over a 5-year period. Additionally, the analysis considered multiple investment phases.

**Figure 4:**
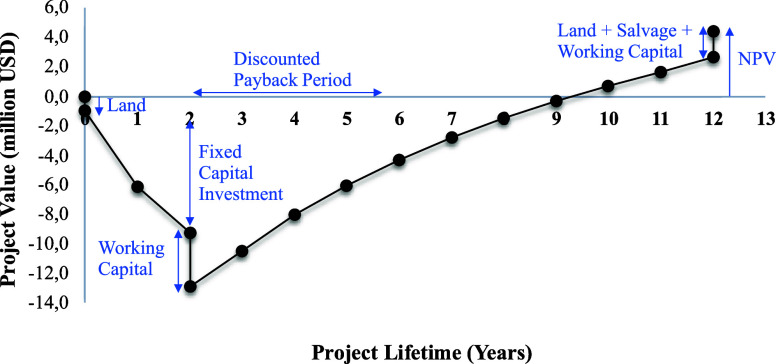
Discounted cash flow diagram.

## 5 Conclusion

Although CHDM can be synthesized via various methods, in this study, an environmentally conscious production route was selected, utilizing waste PET bottles as the feedstock. The proposed process is designed to recycle 7920 tons of waste PET annually, yielding 5000 tons of CHDM per year with a purity of 97.4%. Mass and energy balances of the process were calculated using Aspen Plus software. Equipment sizing, excluding heat exchangers, was performed within Aspen Plus, while the sizing of heat exchangers was carried out using Aspen Exchanger Design & Rating. Following the sizing phase, economic and waste analyses of the proposed process were conducted. The economic evaluation including production cost and profitability analysis was performed using CAPCOST. Based on the simulation, the FCI was calculated as 9.38 million USD, raw material cost as 34.5 million USD, utility cost as 0.14 million USD, operating labor cost as 0.28 million USD, and waste treatment cost as 1.3 million USD. Accordingly, the total production cost was estimated to be 46.7 million USD. For a 10-year project lifetime, the NPV, DCFROR, and discounted payback period (DPBP) were found to be 4.39 million USD, 16.14%, and 3.8 years, respectively. These numbers suggest that CHDM production from waste PET bottles is not only feasible but also economically viable. As a process improvement, the EG formed in the second reactor can be recovered and recycled within the process to improve overall efficiency. In the first reactor, polymerization of EG was assumed to be negligible due to its low extent; however, incorporating these reactions into the model would enable a more accurate and representative simulation of the system. Furthermore, due to the lack of kinetic data in the literature for the second reactor reactions, simulations under varying process conditions could not be performed. Laboratory experiments are suggested to determine the reaction kinetics, which would enhance simulation accuracy. In contrast to most studies reported in the literature, the feed to the second reactor in the present process contains the BHET dimer. It was assumed that only the aromatic rings of the BHET dimer undergo hydrogenation. To enhance the accuracy of the simulation, experimental studies should be conducted to identify the actual hydrogenation products of the BHET dimer and integrate them into the process model.

## Supplementary Material



## Data Availability

The data underlying this study are available in the Supporting Information.

## Notes

Process flow diagram (Figure S1); stream/property tables for the major process streams (Table S1); detailed heat exchanger sizing summaries for E-101, E-102, and E-103 (Tables S2–S4); and detailed economic analysis data, including equipment costs, raw material and product prices, and utility costs (Tables S5–S8) (PDF)
