# Supplementary material for: Process Simulation of 1,4-Cyclohexanedimethanol Production from Waste PETs
Source: ACS Omega. 2026 May 11;11(20):30045–52. doi: 10.1021/acsomega.6c01699 (PMC13216958; doi:10.1021/acsomega.6c01699)
Supplement: Supplementary file 1 [file ao6c01699_si_001.pdf]

Process Simulation of 1,4-Cyclohexanedimethanol Production from Waste PETs

Berk Esin [a], Serhat Sezer [a*], Gülhayat Nasun-Saygılı [a]

[a] Istanbul Technical University, Chemical Engineering Department, 34469, İstanbul, Türkiye

* Corresponding author. E-mail: sezerser@itu.edu.tr

Keywords: Process simulation, Chemical recycling, BHET, Hydrogenation, CHDM

Table of Contents

Figure S1. Aspen Plus Flowsheet (Process Flow Diagram)…………………………2

Table S1. Process Stream Values (Mass & Energy Balance for Key Streams)……...3

Table S2. TEMA Sheet for E-101 (Aspen EDR Design Summary)…………………4

Table S3. TEMA Sheet for E-102 (Aspen EDR Design Summary)…………………7

Table S4. TEMA Sheet for E-103 (Aspen EDR Design Summary)………………..10

Table S5. Equipment Costs…….……………………….……….……….…….……13

Table S6. Raw Material and Product Prices.…………………….…….……..…..13

Table S7. Other Inputs for Economic Analysis…………...………………..……14

Table S8. Utility Types and Their Costs………………………….…..……...14

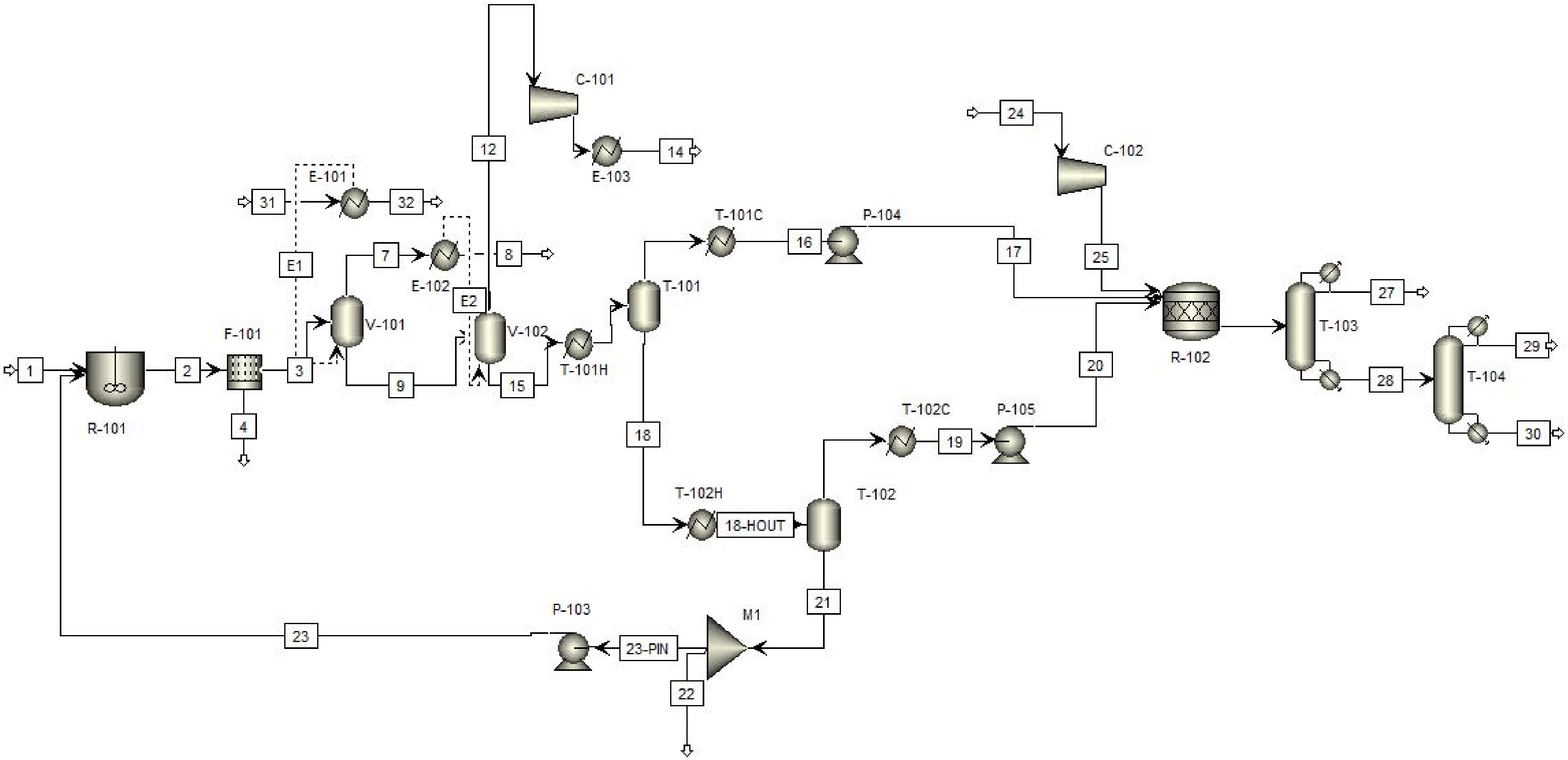

Figure S1. Aspen Plus Flowsheet (Process Flow Diagram)

Table S1. Process Stream Values (Mass & Energy Balance for Key Streams)

Units \ No.		1		2		3	4	5	6	7	8	9	10	11	12	13	14	15		16	
From				R-101		F-101	F-101	P-101	E-101	V-101	E-102	V-101	P-102	E-102	V-102	C-101	E-103	V-102	T-101H	T-101	T-101C
To		R-101		F-101		P-101		E-101	V-101	E-102		P-102	E-102	V-102	C-101	E-103		T-101H	T-101	T-101C	P-104
Phase	-	L	S	L	S	L	S	L	L	G	L	L	L	L	G	G	L	L	L	G	L
Mass Flow	kg/h	5000	1000	6144.48	0.745	6144.48	0.745	1711.08	1711.08	2273.02	2273.02	3871.46	229379.76	229379.76	2358.13	2358.13	2358.13	1513.33	1513.33	806.66	806.66
Volume Flow	L/min	74.5	13.2	103.74	0.011	103.74	0.011			1842880	35.68	58.81			58977800	2696940	37.02	21.46	23.22	11006700	12.50
Temperature	°C	25	25	190	190	190	190	90.1	94.6	90.1	86.6	90.1	74.2	77.2	74.13	239.18	86.83	74.13	205	205	210
Pressure	bar	1.013	1.013	1.013	1.013	1.013	1.013	0.01	0.009	0.01	0.01	0.01	0.01	0.01	0.00031	0.01	0.013	0.000031	0.10022	0.00023	0.00023
Components																					
PET	kg/h	0	1000	0	0.745	0	0.745	0	0	0	0	0	0	0	0	0	0	0	0	0	0
EG	kg/h	5000	0	4686.45	0	4686.45	0	1066.67	1066.67	2273.02	2273.02	2413.43	8383.48	8383.48	2358.12	2358.12	2358.12	55.31	55.31	55.30	55.30
BHET	kg/h	0	0	1308.40	0	1308.40	0	578.28	578.28	1.2E-04	1.2E-04	1308.40	198316.23	198316.23	0.01	0.01	0.01	1308.39	1308.39	736.30	736.30
H_2	kg/h	0	0	0	0	0	0	0	0	0	0	0	0	0	0	0	0	0	0	0	0
CHDM	kg/h	0	0	0	0	0	0	0	0	0	0	0	0	0	0	0	0	0	0	0	0
ETOH*	kg/h	0	0	0	0	0	0	0	0	0	0	0	0	0	0	0	0	0	0	0	0
H_2O	kg/h	0	0	0	0	0	0	0	0	0	0	0	0	0	0	0	0	0	0	0	0
6*	kg/h	0	0	0	0	0	0	0	0	0	0	0	0	0	0	0	0	0	0	0	0
5*	kg/h	0	0	0	0	0	0	0	0	0	0	0	0	0	0	0	0	0	0	0	0
4*	kg/h	0	0	0	0	0	0	0	0	0	0	0	0	0	0	0	0	0	0	0	0
3*	kg/h	0	0	0	0	0	0	0	0	0	0	0	0	0	0	0	0	0	0	0	0
BHET-DIM	kg/h	0	0	149.63	0	0	0	66.13	66.13	1.6E-05	1.6E-05	149.63	22680.05	22680.05	0.0018	0.0018	0.0018	149.63	149.63	15.06	15.06
BHCD	kg/h	0	0	0	0	0	0	0	0	0	0	0	0	0	0	0	0	0	0	0	0
BDIM-AH*	kg/h	0	0	0	0	0	0	0	0	0	0	0	0	0	0	0	0	0	0	0	0

*p-Xylene, Ethyl p-toluate, trans-1,4-dimethylcyclohexane, 4-methylcyclohexanemethanol, Ethanol, $C_{22}H_{34}O_{10}$ are named as 6, 5, 4, 3, ETOH, BDIM-AH in Aspen Plus.

Table S1. Process Stream Values (Mass & Energy Balance for Key Streams) (continued)

Units \ No.		17	18		19		20	21	22	23	24	25	26	27	28	29	30	31	32
From		P-104	T-101	T-102H	T-102	T-102C	P-105	T-102	T-102	P-103		C-102	R-102	T-103	T-103	T-104	T-104		E-101
To		R-102	T-102H	T-102	T-102C	P-105	R-102	P-103		R-101	C-102	R-102	T-103		T-104			E-101	
Phase	-	L	L	L	G	L	L	L	L	L	G	G	L	L	L	L	L	G	L
Mass Flow	kg/h	806.66	706.67	706.67	559.97	559.97	559.97	146.69	1.46	145.22	73.59	73.59	1440.23	738.64	701.39	631.25	70.14	307	307
Volume Flow	L/min	12.55	10.76	10.76	9412580	8.56	8.59	2.22	0.022	2.20	14894.04	1154.38	150.45	12.33	8.16	6.92	0.70	1650.1	6.00
Temperature	°C	216.31	205	208	208	208	214.46	208	208	208.13	25	857.17	260	92.28	265.58	235.61	286.86	158.92	158.92
Pressure	bar	50	0.00023	0.00023	0.00015	0.00015	50	0.00015	0.00015	1.103	1.013	50	50	0.901	0.972	0.405	0.496	6.00	6.00
Components																			
PET	kg/h	0	0	0	0	0	0	0	0	0	0	0	0	0	0	0	0	0	0
EG	kg/h	55.30	0.01	0.01	0.01	0.01	0.01	5.6E-07	5.6E-07	5.6E-07	0	0	602.86	586.90	15.96	15.96	3.2E-06	0	0
BHET	kg/h	736.30	572.09	572.09	509.71	509.71	509.71	62.37	0.62	61.75	0	0	0	0	0	0	0	0	0
H_2	kg/h	0	0	0	0	0	0	0	0	0	73.59	73.59	0.01	5E-49	5.3E-03	8.8E-128	5.3E-03	0	0
CHDM	kg/h	0	0	0	0	0	0	0	0	0	0	0	618.31	0.26	618.05	614.99	3.05	0	0
ETOH*	kg/h	0	0	0	0	0	0	0	0	0	0	0	43.08	43.08	2.4E-04	2.4E-04	8.8E-15	0	0
H_2O	kg/h	0	0	0	0	0	0	0	0	0	0	0	37.55	37.55	2.4E-04	2.4E-04	7.7E-15	307	307
6*	kg/h	0	0	0	0	0	0	0	0	0	0	0	19.15	19.15	1.7E-03	1.7E-03	2.63E-12	0	0
5*	kg/h	0	0	0	0	0	0	0	0	0	0	0	7.40	7.28	0.12	0.12	1.5E-07	0	0
4*	kg/h	0	0	0	0	0	0	0	0	0	0	0	34.84	34.84	3.9E-04	3.9E-04	2.8E-13	0	0
3*	kg/h	0	0	0	0	0	0	0	0	0	0	0	9.95	9.78	0.17	0.17	2.6E-07	0	0
BHET-DIM	kg/h	15.06	134.57	134.57	50.25	50.25	50.25	84.32	0.84	83.47	0	0	0	0	0	0	0	0	0
BHCD	kg/h	0	0	0	0	0	0	0	0	0	0	0	0	0	0	0	0	0	0
BDIM-AH*	kg/h	0	0	0	0	0	0	0	0	0	0	0	67.08	1.9E-20	67.08	5.29E-14	67.08	0	0

*p-Xylene, Ethyl p-toluate, trans-1,4-dimethylcyclohexane, 4-methylcyclohexanemethanol, Ethanol, $C_{22}H_{34}O_{10}$ are named as 6, 5, 4, 3, ETOH, BDIM-AH in Aspen Plus.

Table S2. TEMA Sheet for E-101 (Aspen EDR Design Summary)

Recap of Design

Current selected case: A

		A
**Shell ID**	**mm**	315.93
**Tube length - actual**	**mm**	3048
**Tube length - required**	**mm**	2598.2
**Pressure drop, SS**	**bar**	0.01235
**Pressure drop, TS**	**bar**	0.00051
**Baffle spacing**	**mm**	304.8
**Number of baffles**		9
**Tube passes**		1
**Tube number**		127
**Number of units in series**		1
**Number of units in parallel**		1
**Total price**	**Dollar(US)**	18780
**Program mode**		Design (Sizing)
**Calculation method**		Advanced method
**Area Ratio (dirty)**	*****	1.17
**Film coef overall, SS**	**W/(m2*K)**	11775.1
**Film coef overall, TS**	**W/(m2*K)**	141.4
**Heat load**	**kW**	177.8
**Recap case fully recoverable**		Yes

Table S2. TEMA Sheet for E-101 (Aspen EDR Design Summary) (continued)

TEMA Sheet

1	Company:					
2	Location:					
3	Service of Unit:	Our Reference:				
4	Item No.:	Your Reference:				
5	Date:	Rev No.:	Job No.:			
6	Size: 305 - 3048 mm	Type: BEM Vertical		Connected in: 1 parallel 1 series		
7	Surf/unit(eff.) 22.7 m^2	Shells/unit 1		Surf/shell(eff.) 22.7 m^2		
8	**PERFORMANCE OF ONE UNIT**					
9	Fluid allocation		Shell Side		Tube Side	
10	Fluid name		29		7	
11	Fluid quantity, Total	kg/s	0.0853		0.4753	
12	Vapor (In/Out)	kg/s	0.0853	0	0	0
13	Liquid	kg/s	0	0.0853	0.4753	0.4753
14	Noncondensable	kg/s	0	0	0	0
15						
16	Temperature (In/Out)	°C	158.92	158.84	90.1	94.62
17	Dew / Bubble point	°C	158.92	158.92		
18	Density Vapor/Liquid	kg/m^3	3.1 /	3.09 / 853.02	/ 1108.16	/ 1103.77
19	Viscosity	mPa-s	0.0149 /	0.0149 / 0.169	/ 3.7676	/ 3.3885
20	Molecular wt, Vap		18.02	18.02		
21	Molecular wt, NC					
22	Specific heat	kJ/(kg-K)	1.977 /	1.977 / 4.834	/ 2.309	/ 2.325
23	Thermal conductivity	W/(m-K)	0.0313 /	0.0313 / 0.6839	/ 0.1767	/ 0.176
24	Latent heat	kJ/kg	2085.3	2085.6		
25	Pressure (abs)	bar	6	5.98765	0.01	0.00949
26	Velocity (Mean/Max)	m/s	0.7 / 1.87		0.02 / 0.02	
27	Pressure drop, allow./calc.	bar	0.25855	0.01235	0.00345	0.00051
28	Fouling resistance (min)	m^2-K/W	0		0	0 Ao based
29	Heat exchanged 177.8 kW			MTD (corrected) 66.9 °C		
30	Transfer rate, Service 117		Dirty 137.3	Clean 137.3 W/(m^2-K)		

31	**CONSTRUCTION OF ONE SHELL**				**Sketch**
32			Shell Side	Tube Side	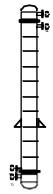
33	Design/Vacuum/test pressure	bar	6.89476 / /	3.44738 / /	
34	Design temperature	°C	198.89	132.22	
35	Number passes per shell		1	1	
36	Corrosion allowance	mm	0	0	
37	Connections	In mm	1 38.1 / -	1 50.8 / -	
38	Size/Rating	Out	1 12.7 / -	1 50.8 / -	
39	Nominal	Intermediate	/ -	/ -	

40	Tube No. 127 OD 19.05 TksAverage 1.65 mm Length 3048 mm Pitch 23.81 mm		
41	Tube type Plain #/m Material SS 316	Tube pattern 30	
42	Shell SS 316 ID 315.93 OD 323.85 mm	Shell cover -	
43	Channel or bonnet SS 316	Channel cover -	
44	Tubesheet-stationary SS 316 -	Tubesheet-floating -	
45	Floating head cover -	Impingement protection None	
46	Baffle-cross SS 316 Type Single segmental Cut(%d) 36.94 Verti	Spacing: c/c 304.8 mm	
47	Baffle-long - Seal Type	Inlet 274.64 mm	
48	Supports-tube U-bend 0 Type		
49	Bypass seal Tube-tubesheet joint Expanded only (2 grooves)(App.A 'i')		
50	Expansion joint - Type None		
51	RhoV2-Inlet nozzle 1360 Bundle entrance 16 Bundle exit 0 kg/(m-s^2)		
52	Gaskets - Shell side - Tube side Flat Metal Jacket Fibe		
53	Floating head -		
54	Code requirements ASME Code Sec VIII Div 1 TEMA class R - refinery service		
55	Weight/Shell 538.6 Filled with water 756 Bundle 332.8 kg		
56	Remarks		
57			
58			

Table S2. TEMA Sheet for E-101 (Aspen EDR Design Summary) (continued)

Overall Summary

No.	Item	Unit	Shell Side In	Shell Side Out	Tube Side In	Tube Side Out	Parameter	Value 1	Value 2	Value 3
1	Size 315.93 X 3048	mm	Type BEM	Ver			Connected in	1 parallel	1 series	
2	Surf/Unit (gross/eff/finned)		23.2 /	22.7 /		m²	Shells/unit	1		
3	Surf/Shell (gross/eff/finned)		23.2 /	22.7 /		m²				
4	Design (Sizing)		**PERFORMANCE OF ONE UNIT**							
5			**Shell Side**		**Tube Side**		**Heat Transfer Parameters**			
6	**Process Data**		**In**	**Out**	**In**	**Out**	Total heat load	kW	177.8	
7	Total flow	kg/s	0.0853		0.4753		Eff. MTD/ 1 pass MTD	°C	66.9 /	66.72
8	Vapor	kg/s	0.0853	0	0	0	Actual/Reqd area ratio - fouled/clean		1.17 /	1.17
9	Liquid	kg/s	0	0.0853	0.4753	0.4753				
10	Noncondensable	kg/s	0		0		**Coef./Resist.**	W/(m²-K)	m²-K/W	%
11	Cond./Evap.	kg/s	0.0853		0		Overall fouled	137.3	0.00728	
12	Temperature	°C	158.92	158.84	90.1	94.62	Overall clean	137.3	0.00728	
13	Dew / Bubble point	°C	158.92	158.92			Tube side film	141.4	0.00707	97.12
14	Quality		1	0	0	0	Tube side fouling		0	0
15	Pressure (abs)	bar	6	5.98765	0.01	0.00949	Tube wall	8003.4	0.00012	1.72
16	DeltaP allow/cal	bar	0.25855	0.01235	0.00345	0.00051	Outside fouling		0	0
17	Velocity	m/s	1.33	0.01	0.02	0.02	Outside film	11775.1	8E-05	1.17
18	**Liquid Properties**						**Shell Side Pressure Drop**		bar	%
19	Density	kg/m³		853.02	1108.16	1103.77	Inlet nozzle		0.01087	87.75
20	Viscosity	mPa-s		0.169	3.7676	3.3885	InletspaceXflow		0.00018	1.44
21	Specific heat	kJ/(kg-K)		4.834	2.309	2.325	Baffle Xflow		0.00031	2.5
22	Therm. cond.	W/(m-K)		0.6839	0.1767	0.176	Baffle window		0.00011	0.9
23	Surface tension	N/m		0.0465			OutletspaceXflow		1E-05	0.1
24	Molecular weight			18.02	86.8	86.8	Outlet nozzle		0.00091	7.31
25	**Vapor Properties**						Intermediate nozzles			
26	Density	kg/m³	3.1	3.09			**Tube Side Pressure Drop**		bar	%
27	Viscosity	mPa-s	0.0149	0.0149			Inlet nozzle		0.00023	45.93
28	Specific heat	kJ/(kg-K)	1.977	1.977			Entering tubes		0	0.16
29	Therm. cond.	W/(m-K)	0.0313	0.0313			Inside tubes		0.00017	33.08
30	Molecular weight		18.02	18.02			Exiting tubes		0	0.23
31	**Two-Phase Properties**						Outlet nozzle		0.0001	20.6
32	Latent heat	kJ/kg	2085.3	2085.6			Intermediate nozzles			
33	**Heat Transfer Parameters**						**Velocity / Rho*V2**	m/s		kg/(m-s²)
34	Reynolds No. vapor		5269	1.08			Shell nozzle inlet	20.95		1360
35	Reynolds No. liquid			464.09	80.31	89.29	Shell bundle Xflow	1.33	0.01	
36	Prandtl No. vapor		0.94	0.94			Shell baffle window	1.87	0.01	
37	Prandtl No. liquid			1.19	49.22	44.76	Shell nozzle outlet	0.54		235
38	**Heat Load**		kW		kW		Shell nozzle interm			
39	Vapor only		0		0			m/s		kg/(m-s²)
40	2-Phase vapor		0		0		Tube nozzle inlet	0.2		43
41	Latent heat		-177.8		0		Tubes	0.02	0.02	
42	2-Phase liquid		0		0		Tube nozzle outlet	0.2		44
43	Liquid only		0		177.8		Tube nozzle interm			

No.	**Tubes**			**Baffles**			**Nozzles: (No./OD)**		**Shell Side**	**Tube Side**
45	Type		Plain	Type	Single segmental					
46	ID/OD	mm	15.75 / 19.05	Number		9	Inlet	mm	1 / 48.26	1 / 60.32
47	Length act/eff	mm	3048 / 2987.7	Cut(%d)	36.94		Outlet		1 / 21.34	1 / 60.32
48	Tube passes		1	Cut orientation		V	Intermediate		/	/
49	Tube No.		127	Spacing: c/c	mm	304.8	Impingement protection		None	
50	Tube pattern		30	Spacing at inlet	mm	274.64				
51	Tube pitch	mm	23.81	Spacing at outlet	mm	274.64				
52	Insert		None							
53	Vibration problem		No / No				RhoV2 violation			No

Table S3. TEMA Sheet for E-102 (Aspen EDR Design Summary)

Recap of Design

Current selected case: D

		D
Shell ID	mm	498.45
Tube length - actual	mm	2438.4
Tube length - required	mm	1979.6
Pressure drop, SS	bar	0.00345
Pressure drop, TS	bar	0.00158
Baffle spacing	mm	603.25
Number of baffles		2
Tube passes		1
Tube number		297
Number of units in series		1
Number of units in parallel		8
Total price	Dollar(US)	254096
Program mode		Design (Sizing)
Calculation method		Advanced method
Area Ratio (dirty)	*	1.23
Film coef overall, SS	W/(m2*K)	316.8
Film coef overall, TS	W/(m2*K)	1932.4
Heat load	kW	635
Recap case fully recoverable		Yes

Table S3. TEMA Sheet for E-102 (Aspen EDR Design Summary) (continued)

TEMA Sheet

1	Company:					
2	Location:					
3	Service of Unit: Our Reference:					
4	Item No.: Your Reference:					
5	Date: Rev No.: Job No.:					
6	Size: 508 - 2438.4 mm Type: BEM Vertical Connected in: 8 parallel 1 series					
7	Surf/unit(eff.) 337.7 m² Shells/unit 8 Surf/shell(eff.) 42.2 m²					
8	**PERFORMANCE OF ONE UNIT**					
9	Fluid allocation		Shell Side		Tube Side	
10	Fluid name		13		5	
11	Fluid quantity, Total	kg/s	63.7166		0.6315	
12	Vapor (In/Out)	kg/s	0	0	0.6315	0.0048
13	Liquid	kg/s	63.7166	63.7166	0	0.6267
14	Noncondensable	kg/s	0	0	0	0
15						
16	Temperature (In/Out)	°C	74.2	77.2	90.1	83.65
17	Dew / Bubble point	°C			86.6	86.6
18	Density Vapor/Liquid	kg/m³	/ 1338.6	/ 1335.66	0.02 /	0.02 / 1064.82
19	Viscosity	mPa-s	/ 11.6268	/ 10.5809	0.0101 /	0.0099 / 2.8509
20	Molecular wt, Vap				62.07	62.07
21	Molecular wt, NC					
22	Specific heat	kJ/(kg-K)	/ 1.826	/ 1.834	1.426 /	1.409 / 2.519
23	Thermal conductivity	W/(m-K)	/ 0.1333	/ 0.1328	0.015 /	0.0144 / 0.259
24	Latent heat	kJ/kg			1000.7	1001.7
25	Pressure (abs)	bar	0.01	0.00655	0.01	0.00842
26	Velocity (Mean/Max)	m/s	0.08 / 0.13		1.17 / 66.37	
27	Pressure drop, allow./calc.	bar	0.00345	0.00345	0.00345	0.00158
28	Fouling resistance (min)	m²-K/W	0		0	0 Ao based
29	Heat exchanged 635 kW MTD (corrected) 8.8 °C					
30	Transfer rate, Service 213.7 Dirty 263.2 Clean 263.2 W/(m²-K)					

31	**CONSTRUCTION OF ONE SHELL**					**Sketch**
32				Shell Side	Tube Side	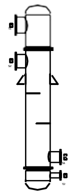
33	Design/Vacuum/test pressure		bar	3.44738 / /	3.44738 / /	
34	Design temperature		°C	115.56	126.67	
35	Number passes per shell			1	1	
36	Corrosion allowance		mm	0	0	
37	Connections	In	mm	1 203.2 / -	1 304.8 / -	
38	Size/Rating	Out		1 254 / -	1 88.9 / -	
39	Nominal	Intermediate		/ -	/ -	
40	Tube No. 297 OD 19.05 TksAverage 1.65 mm Length 2438.4 mm Pitch 23.81 mm					
41	Tube type Plain #/m Material SS 316				Tube pattern 30	
42	Shell SS 316 ID 498.45 OD 508 mm				Shell cover -	
43	Channel or bonnet SS 316				Channel cover -	
44	Tubesheet-stationary SS 316 -				Tubesheet-floating -	
45	Floating head cover -				Impingement protection None	
46	Baffle-cross SS 316 Type Single segmental Cut(%d) 41.73 Hori				Spacing: c/c 603.25 mm	
47	Baffle-long - Seal Type				Inlet 885.82 mm	
48	Supports-tube U-bend 0 Type					
49	Bypass seal Tube-tubesheet joint Expanded only (2 grooves)(App.A 'i')					
50	Expansion joint - Type None					
51	RhoV2-Inlet nozzle 45 Bundle entrance 5 Bundle exit 5 kg/(m-s²)					
52	Gaskets - Shell side - Tube side Flat Metal Jacket Fibe					
53	Floating head -					
54	Code requirements ASME Code Sec VIII Div 1 TEMA class R - refinery service					
55	Weight/Shell 1045.1 Filled with water 1605.4 Bundle 630.6 kg					
56	Remarks					
57						
58						

Table S3. TEMA Sheet for E-102 (Aspen EDR Design Summary) (continued)

Overall Summary

1	Size	498.45	X	2438.4	m m	Type	BEM	Ver	Connected in 8 parallel 1 series
2	Surf/Unit (gross/eff/finned)			346.7	/ 337.7	/		m²	Shells/unit 8
3	Surf/Shell (gross/eff/finned)			43.3	/ 42.2	/		m²	
4	Design (Sizing)				**PERFORMANCE OF ONE UNIT**				

#			Shell Side		Tube Side					
5			**Shell Side**		**Tube Side**		**Heat Transfer Parameters**			
6	**Process Data**		**In**	**Out**	**In**	**Out**	Total heat load		kW	635
7	Total flow	kg/s	63.7166		0.6315		Eff. MTD/ 1 pass MTD		°C 8.8 /	9.47
8	Vapor	kg/s	0	0	0.6315	0.0048	Actual/Reqd area ratio - fouled/clean		1.23 /	1.23
9	Liquid	kg/s	63.7166	63.7166	0	0.6267				
10	Noncondensable	kg/s	0		0		**Coef./Resist.**	W/(m²-K)	m²-K/W	%
11	Cond./Evap.	kg/s	0		0.6267		Overall fouled	263.2	0.0038	
12	Temperature	°C	74.2	77.2	90.1	83.65	Overall clean	263.2	0.0038	
13	Dew / Bubble point	°C			86.6	86.6	Tube side film	1932.4	0.00052	13.62
14	Quality		0	0	1	0.01	Tube side fouling		0	0
15	Pressure (abs)	bar	0.01	0.00655	0.01	0.00842	Tube wall	7959	0.00013	3.31
16	DeltaP allow/cal	bar	0.00345	0.00345	0.00345	0.00158	Outside fouling		0	0
17	Velocity	m/s	0.1	0.1	66.37	0.59	Outside film	316.8	0.00316	83.07
18	**Liquid Properties**						**Shell Side Pressure Drop**		bar	%
19	Density	kg/m³	1338.6	1335.66		1064.82	Inlet nozzle		0.00033	9.67
20	Viscosity	mPa-s	11.6268	10.5809		2.8509	InletspaceXflow		0.001	29.05
21	Specific heat	kJ/(kg-K)	1.826	1.834		2.519	Baffle Xflow		0.00095	27.42
22	Therm. cond.	W/(m-K)	0.1333	0.1328		0.259	Baffle window		0.00012	3.57
23	Surface tension	N/m				0.0428	OutletspaceXflow		0.00093	26.89
24	Molecular weight		228.74	228.74		62.07	Outlet nozzle		0.00012	3.4
25	**Vapor Properties**						Intermediate nozzles			
26	Density	kg/m³			0.02	0.02	**Tube Side Pressure Drop**		bar	%
27	Viscosity	mPa-s			0.0101	0.0099	Inlet nozzle		0.00026	10.13
28	Specific heat	kJ/(kg-K)			1.426	1.409	Entering tubes		0.00024	9.3
29	Therm. cond.	W/(m-K)			0.015	0.0144	Inside tubes		0.0019	74.94
30	Molecular weight				62.07	62.07	Exiting tubes		0	0
31	**Two-Phase Properties**						Outlet nozzle		0.00014	5.63
32	Latent heat	kJ/kg			1000.7	1001.7	Intermediate nozzles			
33	**Heat Transfer Parameters**						**Velocity / Rho*V2**		m/s	kg/(m-s²)
34	Reynolds No. vapor				2126	16.44	Shell nozzle inlet		0.18	45
35	Reynolds No. liquid		215.08	236.34		7.48	Shell bundle Xflow		0.1 0.1	
36	Prandtl No. vapor				0.96	0.97	Shell baffle window		0.13 0.13	
37	Prandtl No. liquid		159.31	146.21		27.72	Shell nozzle outlet		0.12	18
38	**Heat Load**		kW		kW		Shell nozzle interm			
39	Vapor only		0		-3.2				m/s	kg/(m-s²)
40	2-Phase vapor		0		-1.3		Tube nozzle inlet		52.62	57
41	Latent heat		0		-628.2		Tubes		66.37 0.59	
42	2-Phase liquid		0		-2.4		Tube nozzle outlet		5.25	65
43	Liquid only		635		0		Tube nozzle interm			

#									
44	**Tubes**			**Baffles**			**Nozzles: (No./OD)**		
45	Type		Plain	Type	Single segmental			**Shell Side**	**Tube Side**
46	ID/OD	mm 15.75	/ 19.05	Number		2	Inlet	mm 1 / 219.08	1 / 323.85
47	Length act/eff	mm 2438.4	/ 2374.9	Cut(%d)	41.73		Outlet	1 / 273.05	1 / 101.6
48	Tube passes	1		Cut orientation		H	Intermediate	/	/
49	Tube No.	297		Spacing: c/c	mm	603.25	Impingement protection	None	
50	Tube pattern	30		Spacing at inlet	mm	885.82			
51	Tube pitch	mm 23.81		Spacing at outlet	mm	885.82			
52	Insert		None						
53	Vibration problem	No	/ No				RhoV2 violation		No

Table S4. TEMA Sheet for E-103 (Aspen EDR Design Summary)

Recap of Design

Current selected case: **A**

		A
**Shell ID**	**mm**	889
**Tube length - actual**	**mm**	1778
**Tube length - required**	**mm**	101.4
**Pressure drop, SS**	**bar**	0.02633
**Pressure drop, TS**	**bar**	0.00104
**Baffle spacing**	**mm**	596.9
**Number of baffles**		2
**Tube passes**		1
**Tube number**		1168
**Number of units in series**		1
**Number of units in parallel**		2
**Total price**	**Dollar(US)**	163098
**Program mode**		Design (Sizing)
**Calculation method**		Advanced method
**Area Ratio (dirty)**	*****	17.54
**Film coef overall, SS**	**W/(m2*K)**	2175.1
**Film coef overall, TS**	**W/(m2*K)**	2495.1
**Heat load**	**kW**	816.3
**Recap case fully recoverable**		Yes

Table S4. TEMA Sheet for E-103 (Aspen EDR Design Summary) (continued)

TEMA Sheet

1	Company:					
2	Location:					
3	Service of Unit:	Our Reference:				
4	Item No.:	Your Reference:				
5	Date:	Rev No.:	Job No.:			
6	Size: 889 - 1778	mm	Type: BEM Vertical	Connected in: 2 parallel	1 series	
7	Surf/unit(eff.) 235.7	m²	Shells/unit 2	Surf/shell(eff.) 117.8	m²	
8	**PERFORMANCE OF ONE UNIT**					
9	Fluid allocation		Shell Side		Tube Side	
10	Fluid name		Cooling Water		11	
11	Fluid quantity, Total	kg/s	19.7275		0.655	
12	Vapor (In/Out)	kg/s	0	0	0.655	0.0035
13	Liquid	kg/s	19.7275	19.7275	0	0.6515
14	Noncondensable	kg/s	0	0	0	0
15						
16	Temperature (In/Out)	°C	28	38	239.26	84.71
17	Dew / Bubble point	°C			86.6	86.6
18	Density Vapor/Liquid	kg/m³	/ 991.15	/ 981.42	0.01 /	0.02 / 1063.8
19	Viscosity	mPa-s	/ 0.8551	/ 0.6975	0.0142 /	0.01 / 2.7868
20	Molecular wt, Vap				62.07	62.07
21	Molecular wt, NC					
22	Specific heat	kJ/(kg-K)	/ 4.122	/ 4.157	1.796 /	1.412 / 2.523
23	Thermal conductivity	W/(m-K)	/ 0.6105	/ 0.6235	0.0293 /	0.0145 / 0.2591
24	Latent heat	kJ/kg			1000.7	1001.4
25	Pressure (abs)	bar	1.013	0.98667	0.01	0.00896
26	Velocity (Mean/Max)	m/s	0.1 / 0.11		0.82 / 98.79	
27	Pressure drop, allow./calc.	bar	0.20684	0.02633	0.00345	0.00104
28	Fouling resistance (min)	m²-K/W	0		0	0 Ao based
29	Heat exchanged 816.3	kW		MTD (corrected) 59.27	°C	
30	Transfer rate, Service 58.4		Dirty 1025.1	Clean 1025.1	W/(m²-K)	
31	**CONSTRUCTION OF ONE SHELL**				**Sketch**	
32			Shell Side	Tube Side	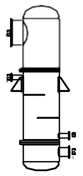	
33	Design/Vacuum/test pressure	bar	3.44738 / /	3.44738 / /		
34	Design temperature	°C	76.67	276.67		
35	Number passes per shell		1	1		
36	Corrosion allowance	mm	0	0		
37	Connections In	mm	1 101.6 / -	1 609.6 / -		
38	Size/Rating Out		1 101.6 / -	1 203.2 / -		
39	Nominal Intermediate		/ -	/ -		
40	Tube No. 1168 OD 19.05 TksAverage 1.65 mm	Length 1778 mm	Pitch 23.81 mm			
41	Tube type Plain	#/m	Material SS 316	Tube pattern 30		
42	Shell SS 316 ID 889 OD 901.7	mm	Shell cover -			
43	Channel or bonnet SS 316		Channel cover -			
44	Tubesheet-stationary SS 316 -		Tubesheet-floating -			
45	Floating head cover -		Impingement protection None			
46	Baffle-cross SS 316	Type Single segmental	Cut(%d) 40.72 Hori	Spacing: c/c 596.9	mm	
47	Baffle-long -	Seal Type		Inlet 544.51	mm	
48	Supports-tube	U-bend 0		Type		
49	Bypass seal		Tube-tubesheet joint	Expanded only (2 grooves)(App.A 'i')		
50	Expansion joint -		Type None			
51	RhoV2-Inlet nozzle 1455	Bundle entrance 60		Bundle exit 60	kg/(m-s²)	
52	Gaskets - Shell side -	Tube side		Flat Metal Jacket Fibe		
53	Floating head -					
54	Code requirements ASME Code Sec VIII Div 1		TEMA class R - refinery service			
55	Weight/Shell 2856	Filled with water 4743.3		Bundle 1841.9	kg	
56	Remarks					
57						
58						

Table S4. TEMA Sheet for E-103 (Aspen EDR Design Summary) (continued)

Overall Summary

#	Item	Unit	Shell Side In	Shell Side Out	Tube Side In	Tube Side Out	Parameter			
1	Size 889 X 1778 mm	Type BEM	Ver				Connected in	2 parallel	1 series	
2	Surf/Unit (gross/eff/finned)		248.6 / 235.7 /			m²	Shells/unit	2		
3	Surf/Shell (gross/eff/finned)		124.3 / 117.8 /			m²				
4	Design (Sizing)			PERFORMANCE OF ONE UNIT						
5			Shell Side		Tube Side		**Heat Transfer Parameters**			
6	**Process Data**		In	Out	In	Out	Total heat load	kW	816.3	
7	Total flow	kg/s	19.7275		0.655		Eff. MTD/ 1 pass MTD	°C	59.27 /	59.57
8	Vapor	kg/s	0	0	0.655	0.0035	Actual/Reqd area ratio - fouled/clean		17.54 /	17.54
9	Liquid	kg/s	19.7275	19.7275	0	0.6515				
10	Noncondensable	kg/s	0		0		**Coef./Resist.**	W/(m²-K)	m²-K/W	%
11	Cond./Evap.	kg/s	0		0.6515		Overall fouled	1025.1	0.00098	
12	Temperature	°C	28	38	239.26	84.71	Overall clean	1025.1	0.00098	
13	Dew / Bubble point	°C			86.6	86.6	Tube side film	2495.1	0.0004	41.08
14	Quality		0	0	1	0.01	Tube side fouling		0	0
15	Pressure (abs)	bar	1.013	0.98667	0.01	0.00896	Tube wall	8695.2	0.00012	11.79
16	DeltaP allow/cal	bar	0.20684	0.02633	0.00345	0.00104	Outside fouling		0	0
17	Velocity	m/s	0.09	0.1	98.79	0.41	Outside film	2175.1	0.00046	47.13
18	**Liquid Properties**						**Shell Side Pressure Drop**		bar	%
19	Density	kg/m³	991.15	981.42		1063.8	Inlet nozzle		0.01422	54.01
20	Viscosity	mPa-s	0.8551	0.6975		2.7868	InletspaceXflow		0.00086	3.25
21	Specific heat	kJ/(kg-K)	4.122	4.157		2.523	Baffle Xflow		0.00035	1.32
22	Therm. cond.	W/(m-K)	0.6105	0.6235		0.2591	Baffle window		5E-05	0.17
23	Surface tension	N/m				0.0427	OutletspaceXflow		0.00081	3.07
24	Molecular weight		18.02	18.02		62.07	Outlet nozzle		0.01005	38.18
25	**Vapor Properties**						Intermediate nozzles			
26	Density	kg/m³			0.01	0.02	**Tube Side Pressure Drop**		bar	%
27	Viscosity	mPa-s			0.0142	0.01	Inlet nozzle		0.00044	16.88
28	Specific heat	kJ/(kg-K)			1.796	1.412	Entering tubes		0.00039	14.96
29	Therm. cond.	W/(m-K)			0.0293	0.0145	Inside tubes		0.00171	65.56
30	Molecular weight				62.07	62.07	Exiting tubes		0	0
31	**Two-Phase Properties**						Outlet nozzle		7E-05	2.6
32	Latent heat	kJ/kg			1000.7	1001.4	Intermediate nozzles			
33	**Heat Transfer Parameters**						**Velocity / Rho*V2**	m/s		kg/(m-s²)
34	Reynolds No. vapor				1596.22	12.19	Shell nozzle inlet	1.21		1455
35	Reynolds No. liquid		2095.12	2568.52		8.09	Shell bundle Xflow	0.09	0.1	
36	Prandtl No. vapor				0.87	0.97	Shell baffle window	0.09	0.09	
37	Prandtl No. liquid		5.77	4.65		27.14	Shell nozzle outlet	1.22		1470
38	**Heat Load**		kW		kW		Shell nozzle interm			
39	Vapor only		0		-161.6			m/s		kg/(m-s²)
40	2-Phase vapor		0		-0.7		Tube nozzle inlet	82.05		98
41	Latent heat		0		-652.8		Tubes	98.79	0.41	
42	2-Phase liquid		0		-1.3		Tube nozzle outlet	2.94		30
43	Liquid only		816.3		0		Tube nozzle interm			

#	**Tubes**	Unit	Value	**Baffles**	Unit	Value	**Nozzles: (No./OD)**	Unit	Shell Side	Tube Side
45	Type		Plain	Type		Single segmental			**Shell Side**	**Tube Side**
46	ID/OD	mm	15.75 / 19.05	Number		2	Inlet	mm	1 / 114.3	1 / 609.6
47	Length act/eff	mm	1778 / 1685.9	Cut(%d)		40.72	Outlet		1 / 114.3	1 / 219.08
48	Tube passes		1	Cut orientation		H	Intermediate		/	/
49	Tube No.		1168	Spacing: c/c	mm	596.9	Impingement protection		None	
50	Tube pattern		30	Spacing at inlet	mm	544.51				
51	Tube pitch	mm	23.81	Spacing at outlet	mm	544.51				
52	Insert		None							
53	Vibration problem		No / No				RhoV2 violation		No	

Table S5. Equipment Costs

Equipment	Total Module Cost (USD)	Grass Roots Cost (USD)
C-101	2,050,000	2,460,000
C-102	2,050,000	2,460,000
E-101	180,000	221,000
E-102	434,000	532,000
E-103	186,000	229,000
F-101	247,000	311,000
P-101	28,900	36,900
P-102	38,500	49,200
P-103	28,900	36,900
P-104	53,000	62,000
P-105	48,900	58,000
T-101	122,000	147,000
T-102	125,000	148,000
T-103	185,000	222,000
T-104	187,000	221,000
R-101	790,000	960,000
R-102	241,000	257,000
V-101	194,000	230,000
V-102	620,000	740,000
-These values have been rounded off to nearest integer.		

Table S6. Raw Material and Product Prices

Material	Classification	Price (USD/kg)	Flowrate	Annual Cost (USD)
PET	Raw Material	0.45*	1000.00	3,600,000
EG	Raw Material	0.70	5000.00	28,000,000
CHDM	Product	(5.00)	614.99	(24,599,760)
EG	Product	(0.70)**	4631.14	(25,934,384)
Wastes	Hazardous Waste	0.20***	810.45	1,296,720
H_2	Raw Material	5.00	73.95	2,943,600
* PET prices have been taken as pretreatment PET prices.<br>** EG removed from evaporators has been evaluated as product.<br>*** Represents the estimated unit cost for waste treatment.				

Table S7. Other Inputs for Economic Analysis

Cost of Land (USD)	1,000,000
Taxation Rate	30%
Annual Interest Rate	10%
Salvage Value (USD)	938,000
Working Capital (USD)	4,420,000
FCI_L	938,000
Project Life After start-up (years)	10
Construction Period (years)	2

Table S8. Utility Types and Their Costs

Equipment	Utility	Actual Usage	Annual Utility Cost (USD)
E-101	Low-Pressure Steam	640 MJ/h	68,010
E-102	7 No. Stream	2290 MJ/h	-
E-103	Cooling Water	2940 MJ/h	8,300
P-101	Electricity	1.29 kW*	622
P-102	Electricity	7.86 kW*	3,800
P-103	Electricity	0.019 kW	9
P-104	Electricity	5.03 kW	2,430
P-105	Electricity	3.44 kW	1,670
T-101	Cooling and Heating	456 MJ/h	63,177
T-102	Cooling and Heating	97 MJ/h	978
*Except P-101 and P-102 actual energy usage values have been taken from Aspen Plus. Since there is no circulation for evaporators available in Aspen Plus, P-101 and P-102 pump powers have been calculated manually.			

P-101 Power Calculation Details

Pipeline length: 12 m
Three 90° elbows in the pipeline
Static head: 5 m (Δz = 5 m)

ΔP = 0.00051 bar = 51 Pa (Value from Aspen Exchanger Design & Rating)

$\rho = 1098.84\ kg/m^3$

$\mu = 0.01392$ Pa.s

m = 0.4753 kg/s = 1711.08 kg/h (Value from Aspen Exchanger Design & Rating)

$$\dot{V} = \frac{1711.08\ \frac{kg}{h}}{1098.84\ \frac{kg}{m^3}} = 1.557\ \frac{m^3}{h}$$

$$H = \frac{\Delta P}{\rho g} + \Delta z + \frac{\Delta \vartheta^2}{2g} + \sum H_f$$

$\frac{\Delta P}{\rho g} = \frac{51}{1098.84 \times 9.81}$ = 0.0047 m

Assumption: ϑ = 2.5 m/s

$$1.557\,\frac{m^3}{h} \times \frac{1h}{3600s} = \frac{\pi}{4} D^2 2.5$$

D = 0.0148 m

Pipe Selection: Based on Table 2.8 [1], a standard pipe of 0.016 × 1 mm (Series 1) is selected. New inner diameter: 0.015 m

$$1.557\,\frac{m^3}{h} \times \frac{1h}{3600s} = \frac{\pi}{4} 0.024^2 \vartheta$$

ϑ_{new} = 2.448 m/s

$$Re = \frac{\rho \vartheta D}{\mu}$$

$$Re = \frac{1098.84 \times 2.448 \times 0.015}{0.01392}$$

Re = 2974.068

From Figure 3.26 (Moody Diagram) [1]: f = 0.046

$$H_f = 0.046\left(\frac{12}{0.015}\right)\left(\frac{2.448^2}{2 \times 9.81}\right)$$

H_f = 11.238 m

From Figure 3.27 [1]: $\zeta_{90^\circ \text{ Elbow}}$ = 0.19

$$h_f = (3)(0.19)\left(\frac{2.448^2}{2 \times 9.81}\right)$$

h_f = 0.17 m

$$\sum H_f = 11.238 + 0.17 = 11.408\, m$$

$$H = 5 + 11.408 + 0.0047 = 16.413\, m$$

$P = \frac{\dot{V}\rho g H}{\eta}$

Assumption: Pump efficiency (η) is taken as 90%.

$$P = \frac{1.557 \times \frac{1}{3600} \times 1098.84 \times 9.81 \times 16.413}{0.9}$$

P = 85.05 W = 0.08505 kW

Based on Table 2.16 [1], a standard motor power of 0.09 kW is selected.

P-102 Power Calculation Details

Assumption:

Pipeline length: 12 m
Three 90° elbows in the pipeline
Static head: 5 m (Δz = 5 m)

ΔP = (0.01 - 0.00031) + (0.00158) = 0.01127 bar = 1127 Pa (Value from Aspen Exchanger Design & Rating)

$\rho = 1177.98$ kg/m^3

$\mu = 0.134$ Pa.s

m = 63.7166 kg/s = 229379.26 kg/h (Value from Aspen Exchanger Design & Rating)

$\dot{V} = \frac{229379.26 \frac{kg}{h}}{1098.84 \frac{kg}{m^3}} = 194.7233 \frac{m^3}{h}$

$H = \frac{\Delta P}{\rho g} + \Delta z + \frac{\Delta \vartheta^2}{2g} + \sum H_f$

$\frac{\Delta P}{\rho g} = \frac{1127}{1177.98 \times 9.81}$ = 0.0975 m

Assumption: $\vartheta = 2.5$ m/s

$194.7233 \frac{m^3}{saat} \times \frac{1h}{3600s} = \frac{\pi}{4} D^2 2.5$

D = 0.165975 m

Pipe Selection: Based on Table 2.8 [1], a standard pipe of 0.1683 × 1.6 mm (Series 1) is selected.

New inner diameter: 0.1667 m

$194.7233\,\frac{m^3}{h} \times \frac{1h}{3600s} = \frac{\pi}{4}0.1^2\vartheta$

$\vartheta_{new} = 2.478$ m/s

$Re = \frac{\rho\vartheta D}{\mu}$

$Re = \frac{1177.98\times2.478\times0.1667}{0.134}$

Re = 3648.821

From Figure 3.26 (Moody Diagram) [1]: f = 0.042

$H_f = 0.042(\frac{12}{0.1667})(\frac{2.478^2}{2\times9.81})$

H_f= 0.947 m

From Figure 3.27 [1]: $\zeta_{90°\ Elbow}$= 0.15

$h_f = (3)(0.15)\left(\frac{2.478^2}{2\times9.81}\right)$

h_f= 0.141 m

$\sum H_f\ = 0.947 + 0.141 = 1.088\,m$

$H = 5 + 1.088 + 0.0975 = 6.1855\,m$

$P = \frac{\dot{V}\,\rho g H}{\eta}$

Assumption: Pump efficiency (η) is taken as 90%.

$$P = \frac{194.7233 \times \frac{1}{3600} \times 11177.98 \times 9.81 \times 6.1855}{0.9}$$

P = 4295.454 W = 4.295 kW

Based on Table 2.16 [1], a standard motor power of 5.5 kW is selected.

References

[1] Bulutçu N., Gürbüz H. “Kimya Mühendisliğinde Tasarım” Istanbul: Istanbul Technical University, 2017.
